# TH1 and TH2 cytokine data in insulin secretagogues users newly diagnosed with breast cancer

**DOI:** 10.1016/j.dib.2017.02.044

**Published:** 2017-02-22

**Authors:** Zachary A.P. Wintrob, Jeffrey P. Hammel, George K. Nimako, Dan P. Gaile, Alan Forrest, Alice C. Ceacareanu

**Affiliations:** a State University of New York at Buffalo, Dept. of Pharmacy Practice, NYS Center of Excellence in Bioinformatics and Life Sciences, 701 Ellicott Street, Buffalo, NY 14203, United States; b Cleveland Clinic, Dept. of Biostatistics and Epidemiology, 9500 Euclid Ave., Cleveland, OH 44195, United States; c State University of New York at Buffalo, Dept. of Biostatistics, 718 Kimball Tower, Buffalo, NY 14214, United States; d The UNC Eshelman School of Pharmacy, Division of Pharmacotherapy and Experimental Therapeutics, Campus Box 7569, Chapel Hill, NC 27599, United States; e Roswell Park Cancer Institute, Dept. of Pharmacy Services, Elm & Carlton Streets, Buffalo, NY 14263, United States

**Keywords:** Type 1T helper cytokine, Type 2T helper cytokine, TH1 cytokine, TH2 cytokine, Cytokine, Secretagogue, Breast cancer, Diabetes, Cancer outcomes, Cancer prognosis

## Abstract

Stimulation of insulin production by insulin secretagogue use may impact T helper cells’ cytokine production. This dataset presents the relationship between baseline insulin secretagogues use in women diagnosed with breast cancer and type 2 diabetes mellitus, the T-helper 1 and 2 produced cytokine profiles at the time of breast cancer diagnosis, and subsequent cancer outcomes. A Pearson correlation analysis evaluating the relationship between T-helper cytokines stratified by of insulin secretagogues use and controls is also provided.

**Specifications Table**

**Table:** 

Subject area	Clinical and Translational Research
More specific subject area	Biomarker Research, Cancer Epidemiology
Type of data	Tables
How data was acquired	Tumor registry query was followed by vital status ascertainment, and medical records review
	Luminex®-based quantitation from plasma samples was conducted for the following T-helper 1 and T-helper 2 cytokines: Interleukine-2, soluble interleukine-2 receptor α, interleukine-12 subunit p40, interleukine-12 subunit p70, interferon α 2, interferon γ, chemokine ligand 10 (interferon gamma-induced protein 10), chemokine ligand 9 (monokine-induced by interferon γ), chemokine ligand 8 (interleukine-8) interleukine-5, interleukine-10, and interleukine-13.
	A Luminex®200^TM^ instrument with Xponent 3.1 software was used to acquire all data
Data format	Analyzed
Experimental factors	T-helper 1 and 2 produced cytokines were determined from the corresponding plasma samples collected at the time of breast cancer diagnosis
Experimental features	The dataset included 97 adult females with diabetes mellitus and newly diagnosed breast cancer (cases) and 194 matched controls (breast cancer only). Clinical and treatment history were evaluated in relationship with cancer outcomes and factor-helper 1 and 2 produced cytokine profiles. A cytokine correlation analysis was also performed.
Data source location	United States, Buffalo, NY - 42° 53׳ 50.3592"N; 78° 52׳ 2.658"W
Data accessibility	The data is with this article

**Value of the data**

• This dataset represents the observed relationship between insulin secretagogues use, circulating T-helper 1 and 2 produced cytokines at breast cancer diagnosis and cancer outcomes
• Presented data has the potential to guide future research exploring the potential use of insulin secretagogues in the modulation of type 1 and type 2 immunity
• Our observations can assist further research exploring the relationship between insulin secretagogues use and T-helper-driven signaling in the occurrence of breast cancer.

## 1 Data

Reported data represents the observed association between pre-existing use of injectable insulin before breast cancer diagnosis and the T-helper 1 and 2 produced cytokine profiles upon cancer diagnosis in women with both breast cancer and diabetes mellitus (Table 1). Data in Table 2 includes the observed correlations between T-helper 1 and 2 cytokines stratified by diabetes mellitus pharmacotherapy and controls.

## 2 Experimental design, materials and methods

Evaluation of the association between profiles of T-helper 1 and 2 produced cytokines, injectable insulin use and BC outcomes was carried out under two protocols approved by both Roswell Park Cancer Institute (EDR154409 and NHR009010) and the State University of New York at Buffalo (PHP0840409E). Demographic and clinical patient information was linked with cancer outcomes and profiles of T-helper 1 and 2 produced cytokines of corresponding plasma specimen harvested at BC diagnosis and banked in the Roswell Park Cancer Institute Data Bank and Bio-Repository.

### 2.1 Study population

All incident breast cancer cases diagnosed at Roswell Park Cancer Institute (01/01/2003-12/31/2009) were considered for inclusion (*n*=2194). Medical and pharmacotherapy history were used to determine the baseline presence of diabetes.

### 2.2 Inclusion and exclusion criteria

All adult women with pre-existing diabetes at breast cancer diagnosis having available banked treatment-naïve plasma specimens (blood collected prior to initiation of any cancer-related therapy - surgery, radiation or pharmacotherapy) in the Institute׳s Data Bank and Bio-Repository were included.

Subjects were excluded if they had prior cancer history or unclear date of diagnosis, incomplete clinical records, type 1 or unclear diabetes status. For a specific breakdown of excluded subjects, please see the original research article by Wintrob et al. [1].

A total of 97 female subjects with breast cancer and baseline diabetes mellitus were eligible for inclusion in this analysis.

### 2.3 Control-matching approach

Each of the 97 adult female subjects with breast cancer and diabetes mellitus (defined as “cases”) was matched with two other female subjects diagnosed with breast cancer, but without baseline diabetes mellitus (defined as “controls”). The following matching criteria were used: age at diagnosis, body mass index category, ethnicity, menopausal status and tumor stage (as per the American Joint Committee on Cancer). Some matching limitations applied [1].

### 2.4 Demographic and clinical data collection

Clinical and treatment history was documented as previously described [1]. Vital status was obtained from the Institute׳s Tumor Registry, a database updated biannually with data obtained from the National Comprehensive Cancer Networks’ Oncology Outcomes Database. Outcomes of interest were breast cancer recurrence and/or death.

### 2.5 Plasma specimen storage and retrieval

All the plasma specimens retrieved from long-term storage were individually aliquoted in color coded vials labeled with unique, subject specific barcodes. Overall duration of freezing time was accounted for all matched controls ensuring that the case and matched control specimens had similar overall storage conditions. Only two instances of freeze-thaw were allowed between biobank retrieval and biomarker analyses: aliquoting procedure step and actual assay.

### 2.6 Luminex® assays

A total of 12 biomarkers - interleukine-2, soluble interleukine-2 receptor α, interleukine-12 subunit p40, interleukine-12 subunit p70, interferon α 2, interferon γ, chemokine ligand 10 (interferon gamma-induced protein 10), chemokine ligand 9 (monokine-induced by interferon γ), chemokine ligand 8 (interleukine-8), interleukine-5, interleukine-10, and interleukine-13 - were quantified according to the manufacturer protocol. The Luminex® HCYTOMAG-60K panel (Millipore Corporation, Billerica, MA) was used in this study.

### 2.7 Biomarker-pharmacotherapy association analysis

Biomarker cut-point optimization was performed for each analyzed biomarker. Biomarker levels constituted the continuous independent variable that was subdivided into two groups that optimized the log rank test among all possible cut-point selections yielding a minimum of 10 patients in any resulting group. Quartiles were also constructed. The resultant biomarker categories were then tested for association with type 2 diabetes mellitus therapy and controls by Fisher׳s exact test. The continuous biomarker levels were also tested for association with diabetes therapy and controls across groups by the Kruskall-Wallis test and pairwise by the Wilcoxon rank sum. Multivariate adjustments were performed accounting for age, tumor stage, body mass index, estrogen receptor status, and cumulative comorbidity. The biomarker analysis was performed using R Version 2.15.3. Please see the original article for an illustration of the analysis workflow [1].

Correlations between biomarkers stratified by type 2 diabetes mellitus pharmacotherapy and controls were assessed by the Pearson method. Correlation models were constructed both with and without adjustment for age, body mass index, and the combined comorbidity index. Correlation analyses were performed using SAS Version 9.4.

## Funding sources

This research was funded by the following grant awards: Wadsworth Foundation Peter Rowley Breast Cancer Grant awarded to A.C.C. (UB Grant number 55705, Contract CO26588).

## Figures and Tables

**Table 1:** T-Helper 1 and 2 produced cytokines’ associations with secretagogue use.

Biomarker	Biomarker Grouping	Concentration (ng/ml)	Control	No Secretagogue	Any Secretagogue	Unadjusted p-value (MVP)			
						p^1^	p^2^	p^3^	Global Tesr
IL-2 (pg/ml)	Median	–	1.60	1.60	1.60	0.420	0.760	0.400	0.650
	(25th–75th)		(1.60–3.2)	(1.60–3.46)	(1.60–3.20)	(0.100)	(0.970)	(0.300)	(0.170)
	OS-Based	0.10 to 34.18	189 (97.4%)	43 (91.5%)	49 (98.0%)	0.080	1.000	0.200	0.140
	Optimization	**35.37 to 516.64**	5 (2.6%)	4 (8.5%)	1 (2.0%)	(0.080)	(0.780)	(0.080)	(0.180)
	DFS-Based	**0.10 to 1.94**	131 (67.5%)	29 (61.7%)	34 (68.0%)	0.450	0.950	0.520	0.730
	Optimization	1.99 to 516.64	63 (32.5%)	18 (38.3%)	16 (32.0%)	(0.440)	(0.660)	(0.510)	(0.660)
									
sIL-2Rα (pg/ml)	Median	–	3.20	6.38	12.07	0.430	0.240	0.880	0.430
	(25th–75th)		(1.60–47.32)	(1.60–98.14)	(1.60–60.42)	(0.100)	(0.630)	(0.230)	(0.210)
	Quartiles	0.00 to 1.60	84 (43.3%)	20 (42.6%)	16 (32.0%)	0.270	0.460	0.270	0.350
		1.70 to 7.00	16 (8.2%)	4 (8.5%)	6 (12.0%)				
		7.12 to 57.42	50 (25.8%)	7 (14.9%)	15 (30.0%)				
		57.68 to ALQ	44 (22.7%)	16 (34.0%)	13 (26.0%)				
	OS-Based	0.00 to 63.34	155 (79.9%)	32 (68.1%)	37 (74.0%)	0.080	0.370	0.520	0.190
	Optimization	**63.37 to ALQ**⁎	39 (20.1%)	15 (31.9%)	13 (26.0%)	(0.070)	(0.070)	(0.900)	(0.100)
	DFS-Based	0.00 to 62.50	153 (78.9%)	32 (68.1%)	37 (74.0%)	0.120	0.460	0.520	0.270
	Optimization	**62.65 to ALQ**⁎	41 (21.1%)	15 (31.9%)	13 (26.0%)	(0.120)	(0.100)	(0.900)	(0.160)
									
IL-12p40 (pg/ml)	Median	–	8.16	16.02	10.10	0.110	0.430	0.400	0.230
	(25th–75th)		(1.75–30.81)	(4.59–41.28)	(3.39–28.42)	(0.090)	(0.560)	(0.110)	(0.180)
	Quartiles	1.25 to 3.20	74 (38.1%)	11 (23.4%)	13 (26.0%)	0.230	0.160	0.560	0.190
		3.94 to 9.74	29 (14.9%)	7 (14.9%)	12 (24.0%)				
		9.94 to 30.67	42 (21.6%)	15 (31.9%)	15 (30.0%)				
		30.92 to 2045.71	49 (25.3%)	14 (29.8%)	10 (20.0%)				
	OS-Based	1.25 to 3.12	53 (27.3%)	8 (17.0%)	9 (18.0%)	0.150	0.180	0.900	0.180
	Optimization	**3.20 to 2045.71**	141 (72.7%)	39 (83.0%)	41 (82.0%)	(0.210)	(0.210)	(0.480)	(0.320)
	DFS-Based	1.25 to 3.12	53 (27.3%)	8 (17.0%)	9 (18.0%)	0.150	0.180	0.900	0.180
	Optimization	**3.20 to 2045.71**⁎	141 (72.7%)	39 (83.0%)	41 (82.0%)	(0.210)	(0.210)	(0.480)	(0.320)
									
IL-12p70 (pg/ml)	Median	–	1.60	3.20	2.12	0.013	0.440	0.190	0.047
	(25th–75th)		(1.60–3.20)	(1.60–7.06)	(1.60–4.40)	(0.023)	(0.980)	(0.270)	(0.053)
	OS-Based	**0.10 to 0.59**	5 (2.6%)	2 (4.3%)	4 (8.0%)	0.620	0.090	0.680	0.140
	Optimization	0.70 to 2510.07	189 (97.4%)	45 (95.7%)	46 (92.0%)	(0.460)	(0.190)	(0.740)	(0.450)
	DFS-Based	0.10 to 2.20	120 (61.9%)	20 (42.6%)	25 (50.0%)	0.018	0.130	0.460	0.033
	Optimization	**2.28 to 2510.07**	74 (38.1%)	27 (57.4%)	25 (50.0%)	(0.033)	(0.380)	(0.420)	(0.100)
									
IFN-α2 (pg/ml)	Median	–	7.24	7.39	8.00	0.460	0.300	0.980	0.510
	(25th–75th)		(3.20–13.61)	(3.20–22.78)	(3.87–16.94)	(0.230)	(0.830)	(0.270)	(0.410)
	Quartiles	0.61 to 1.60	56 (28.9%)	15 (31.9%)	12 (24.0%)	0.390	0.790	0.680	0.710
		3.47 to 7.40	42 (21.6%)	9 (19.1%)	12 (24.0%)				
		7.43 to 15.15	52 (26.8%)	8 (17.0%)	12 (24.0%)				
		15.32 to 1880.18	44 (22.7%)	15 (31.9%)	14 (28.0%)				
	OS-Based	0.61 to 4.18	63 (32.5%)	17 (36.2%)	13 (26.0%)	0.630	0.380	0.280	0.540
	Optimization	**4.18 to 1880.18**⁎	131 (67.5%)	30 (63.8%)	37 (74.0%)	(0.600)	(0.990)	(0.430)	(0.720)
	DFS-Based	0.61 to 2.66	29 (14.9%)	7 (14.9%)	3 (6.0%)	0.990	0.110	0.190	0.240
	Optimization	**2.93 to 1880.18**	165 (85.1%)	40 (85.1%)	47 (94.0%)	(0.600)	(0.047)	(0.130)	(0.100)
									
IFN-γ (pg/ml)	Median	–	13.32	11.26	8.53	0.620	0.140	0.550	0.350
	(25th–75th)		(4.70–36.30)	(3.20–42.84)	(2.80–34.28)	(0.860)	(0.420)	(0.450)	(0.770)
	Quartiles	0.07 to 3.86	42 (21.6%)	13 (27.7%)	18 (36.0%)	0.300	0.120	0.780	0.200
		4.03 to 12.43	50 (25.8%)	11 (23.4%)	12 (24.0%)				
		12.55 to 37.33	56 (28.9%)	8 (17.0%)	8 (16.0%)				
		38.74 to 646.43	46 (23.7%)	15 (31.9%)	12 (24.0%)				
	OS-Based	0.07 to 230.77	188 (96.9%)	44 (93.6%)	49 (98.0%)	0.380	1.000	0.350	0.550
	Optimization	**376.09 to 646.43**	6 (3.1%)	3 (6.4%)	1 (2.0%)	(0.350)	(0.840)	(0.150)	(0.490)
	DFS-Based	0.07 to 187.14	187 (96.4%)	43 (91.5%)	49 (98.0%)	0.230	1.000	0.200	0.250
	Optimization	**206.34 to 646.43**⁎	7 (3.6%)	4 (8.5%)	1 (2.0%)	(0.250)	(0.690)	(0.080)	(0.300)
									
CXCL-10 (IP-10, pg/ml)	Median	–	488	440	470	0.650	0.680	0.910	0.850
	(25th–75th)		(347–814)	(338–728)	(355–662)	(0.990)	(0.210)	(0.170)	(0.350)
	Quartiles	1.6 to 344.8	48 (24.7%)	13 (27.7%)	12 (24.0%)	0.960	0.920	0.950	0.990
		346.1 to 484.3	48 (24.7%)	11 (23.4%)	14 (28.0%)				
		484.5 to 748.4	47 (24.2%)	12 (25.5%)	13 (26.0%)				
		751.0 to 3745.0	51 (26.3%)	11 (23.4%)	11 (22.0%)				
	OS-Based	1.6 to 428.3	81 (41.8%)	22 (46.8%)	21 (42.0%)	0.530	0.970	0.630	0.820
	Optimization	**428.9 to 3745.0**⁎	113 (58.2%)	25 (53.2%)	29 (58.0%)	(0.390)	(0.910)	(0.820)	(0.800)
	DFS-Based	1.6 to 549.1	114 (58.8%)	27 (57.4%)	31 (62.0%)	0.870	0.680	0.650	0.890
	Optimization	**549.1 to 3745.0**⁎	80 (41.2%)	20 (42.6%)	19 (38.0%)	(0.830)	(0.440)	(0.500)	(0.680)
									
CXCL-9 (MIG, pg/ml)	Median	–	274	148	227	0.420	0.730	0.550	0.690
	(25th–75th)		(119–504)	(67–637)	(120–509)	(0.220)	(0.270)	(0.840)	(0.410)
	Quartiles	1.9 to 103.9	46 (23.7%)	17 (36.2%)	10 (20.0%)	0.090	0.600	0.200	0.190
		104.9 to 263.1	47 (24.2%)	10 (21.3%)	16 (32.0%)				
		264.3 to 512.2	55 (28.4%)	6 (12.8%)	11 (22.0%)				
		519.4 to 2691.0	46 (23.7%)	14 (29.8%)	13 (26.0%)				
	OS-Based	1.9 to 120.1	49 (25.3%)	21 (44.7%)	13 (26.0%)	0.010	0.910	0.060	0.027
	Optimization	**121.4 to 2691.0**⁎	145 (74.7%)	26 (55.3%)	37 (74.0%)	(0.002)	(0.470)	(0.120)	(0.013)
	DFS-Based	1.9 to 120.1	49 (25.3%)	21 (44.7%)	13 (26.0%)	0.010	0.910	0.060	0.027
	Optimization	**121.4 to 2691.0**⁎	145 (74.7%)	26 (55.3%)	37 (74.0%)	(0.002)	(0.470)	(0.120)	(0.013)
									
CXCL-8 (IL-8, pg/ml)	Median	–	4.44	6.07	5.72	0.008	0.003	0.880	0.001
	(25th–75th)		(2.50–6.86)	(3.90–9.12)	(4.43–8.35)	(0.090)	(0.090)	(0.960)	(0.090)
	Quartiles	0.36 to 3.07	61 (31.4%)	6 (12.8%)	7 (14.0%)	0.018	0.070	0.920	0.022
		3.15 to 4.89	47 (24.2%)	12 (25.5%)	13 (26.0%)				
		4.91 to 7.53	47 (24.2%)	11 (23.4%)	14 (28.0%)				
		7.68 to 74.69	39 (20.1%)	18 (38.3%)	16 (32.0%)				
	OS-Based	0.36 to 17.15	187 (96.4%)	45 (95.7%)	49 (98.0%)	0.690	1.000	0.610	0.800
	Optimization	**19.66 to 74.69**⁎	7 (3.6%)	2 (4.3%)	1 (2.0%)	(0.900)	(0.740)	(0.270)	(0.830)
	DFS-Based	0.36 to 17.15	187 (96.4%)	45 (95.7%)	49 (98.0%)	0.690	1.000	0.610	0.800
	Optimization	**19.66 to 74.69**⁎	7 (3.6%)	2 (4.3%)	1 (2.0%)	(0.900)	(1.000)	(0.270)	(0.830)
									
IL-5 (pg/ml)	Median, ng/ml	–	0.48	0.45	0.35	0.610	0.011	0.160	0.043
	(25th–75th)		(0.35–0.77)	(0.3–0.77)	(0.3–0.57)	(0.680)	(0.320)	(0.100)	(0.320)
	Quartiles	0.08 to 0.30	38 (19.6%)	17 (36.2%)	21 (42.0%)	0.017	0.008	0.230	0.005
		0.35 to 0.48	76 (39.2%)	8 (17.0%)	14 (28.0%)				
		0.57 to 0.77	49 (25.3%)	14 (29.8%)	7 (14.0%)				
		0.85 to 118	31 (16.0%)	8 (17.0%)	8 (16.0%)				
	OS-Based	0.08 to 0.38	66 (34.0%)	20 (42.6%)	26 (52.0%)	0.270	0.021	0.350	0.054
	Optimization	**0.45 to 118**⁎	128 (66.0%)	27 (57.4%)	24 (48.0%)	(0.250)	(0.170)	(0.540)	(0.180)
	DFS-Based	0.08 to 0.38	66 (34.0%)	20 (42.6%)	26 (52.0%)	0.270	0.021	0.350	0.054
	Optimization	**0.45 to 118**	128 (66.0%)	27 (57.4%)	24 (48.0%)	(0.0250)	(0.170)	(0.540)	(0.180)
									
IL-10 (pg/ml)	Median, ng/ml	–	1.60	3.20	1.95	0.220	0.890	0.290	0.440
	(25th–75th)		(1.60–6.59)	(1.60–12.91)	(1.60–4.70)	(0.150)	(0.770)	(0.210)	(0.230)
	Quartiles	0.18 to 1.6	100 (51.5%)	19 (40.4%)	22 (44.0%)	0.330	0.190	0.110	0.180
		1.61 to 1.78	3 (1.5%)	0 (0%)	2 (4.0%)				
		1.82 to 8.80	44 (22.7%)	11 (23.4%)	17 (34.0%)				
		8.96 to 1197.53	47 (24.2%)	17 (36.2%)	9 (18.0%)				
	OS-Based	0.18 to 3.20	114 (58.8%)	21 (44.7%)	28 (56.0%)	0.080	0.720	0.270	0.220
	Optimization	**3.20 to 1197. 53**⁎	80 (41.2%)	26 (55.3%)	22 (44.0%)	(0.080)	(0.600)	(0.360)	(0.200)
	DFS-Based	0.18 to 1.90	105 (54.1%)	19 (40.4%)	25 (50.0%)	0.090	0.600	0.340	0.240
	Optimization	**2.00 to 1197. 53**⁎	89 (45.9%)	28 (59.6%)	25 (50.0%)	(0.080)	(0.510)	(0.380)	(0.220)
									
IL-13 (pg/ml)	Median, ng/ml	–	1.60	1.60	1.60	0.810	0.290	0.590	0.580
	(25th–75th)		(1.60–4.49)	(1.60–4.38)	(1.60–3.13)	(0.760)	(0.330)	(0.140)	(0.520)
	OS-Based	0.00 to 1.55	24 (12.4%)	7 (14.9%)	8 (16.0%)	0.640	0.500	0.880	0.760
	Optimization	**1.60 to 1239.25**⁎	170 (87.6%)	40 (85.1%)	42 (84.0%)	(0.450)	(0.410)	(0.550)	(0.570)
	DFS-Based	0.00 to 1.01	19 (9.8%)	5 (10.6%)	5 (10.0%)	0.790	1.000	1.000	0.960
	Optimization	**1.16 to 1239.25**	175 (90.2%)	42 (89.4%)	45 (90.0%)	(0.720)	(0.970)	(0.620)	(0.900)

⁎ Overall survival (OS)- and disease-free survival (DFS)-optimized biomarker ranges associated with poorer outcomes are represented in bold. ALQ=above limit of quantitation. MVP= p-value of the multivariate adjusted analysis. Interleukine-2, IL-2; soluble interleukine-2 receptor α, sIL-2Rα; interleukine-12 subunit p40, IL-12p40; interleukine-12 subunit p70, IL-12p70; interferon α 2, IFN-α2; interferon γ, IFN-γ; chemokine ligand 10, CXCL-10 (interferon gamma-induced protein 10, IP-10); chemokine ligand 9, CXCL-9 (monokine-induced by interferon γ, MIG); chemokine ligand 8, CXCL-8 (interleukine-8, IL-8); interleukine-5, IL-5; interleukine-10, IL-10; interleukine-13, IL-13.

**Table 2:** T-Helper 1 and 2 produced cytokines’ correlations by secretagogue use.








